# Placenta-targeted Treatment Strategies for Preeclampsia and Fetal Growth Restriction: An Opportunity and Major Challenge

**DOI:** 10.1007/s12015-024-10739-x

**Published:** 2024-05-30

**Authors:** Jianjian Cui, Zejun Yang, Ruilin Ma, Wencong He, Hui Tao, Ya’nan Li, Yin Zhao

**Affiliations:** 1 Department of Obstetrics and Gynecology, Union Hospital, Tongji Medical College, Huazhong University of Science and Technology, No. 1277 Jiefang Avenue, Wuhan, 430022 China; 2 Shenzhen Huazhong University of Science and Technology Research Institute, Shenzhen, 518000 China

**Keywords:** Targeted treatment, Targeted delivery stategy, Placenta dysfunction, MSCs, Tumor homing peptide, Nanoparticles

## Abstract

The placenta plays a crucial role in maintaining normal pregnancy. The failure of spiral artery remodeling (SAR) is a key factor leading to placental ischemia and poor perfusion which is strongly associated with obstetric diseases, including preeclampsia (PE) and fetal growth restriction (FGR). Existing interventions for PE and FGR are limited and termination of pregnancy is inevitable when the maternal or fetus condition deteriorates. Considering the safety of the mother and fetus, treatments that may penetrate the placental barrier and harm the fetus are not accepted. Developing targeted treatment strategies for these conditions is urgent and necessary. With the proven efficacy of targeted therapy in treating conditions such as endometrial cancer and trophoblastic tumors, research on placental dysfunction continues to deepen. This article reviews the studies on placenta-targeted treatment and drug delivery strategies, summarizes the characteristics proposes corresponding improvement measures in targeted treatment, provides solutions for existing problems, and makes suggestions for future studies.

**Graphical abstract:**

**Figure:**
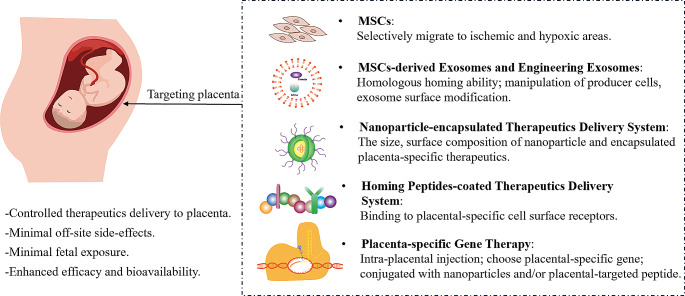
Targeted treatment and therapeutics delivery methods, there are several kinds of placenta targeted methods and those benefits for Obstetric outcomes. Abbreviations: MSCs, mesenchymal stem/stromal cells

## Introduction

**Fig. 1:**
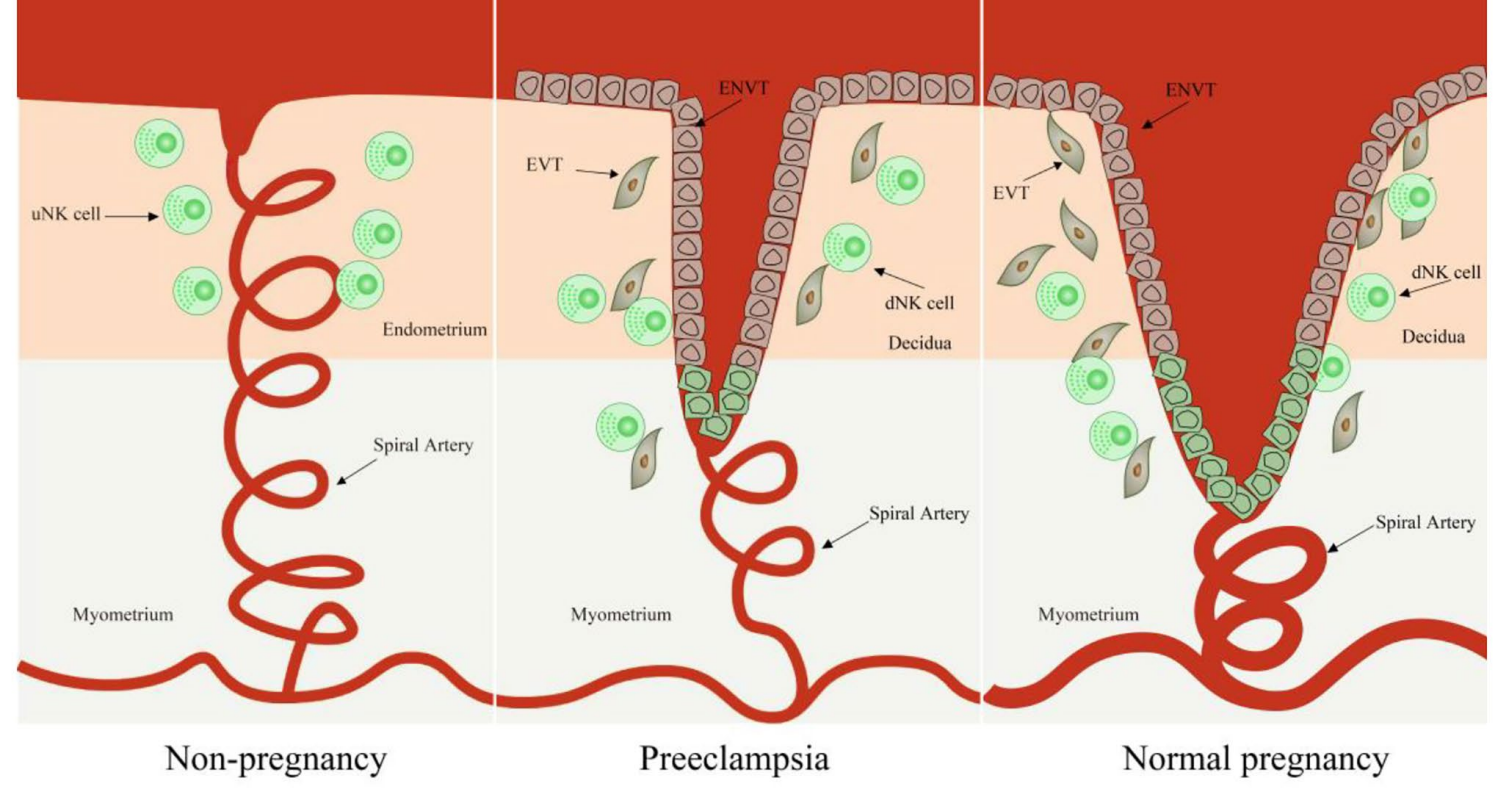
The spiral uterine artery changes in pregnancy and preeclampsia. During normal pregnancy, the spiral artery runs in a spiral shape within the functional layer, forming a capillary network and blood sinuses when it reaches the superficial layer of the functional layer, it then merges into small veins and merges through the muscular layer to form the uterine artery; in preeclampsia, the spiral artery recasting disorder, shows shallow implantation of placenta vessels. Abbreviations: uNK cell: uterine natural killer cell, dNK cell: decidua natural killer cell, EVT: extravillous trophoblast, ENVT: endovascular trophoblast

The placenta is a vital organ that plays a critical role in providing nutrients, eliminating waste products, and shielding the fetus from harmful substances in the maternal circulation [1]. Trophoblasts, the primary cells of the placenta, govern its formation and function [2]. Dysfunctional development of the placenta is a fundamental cause of various pregnancy complications, such as preeclampsia (PE) and fetal growth restriction (FGR). PE stands out as one of the most serious pregnancy complications, with profound consequences for both the mother and the fetus. The primary pathophysiology of PE involves the failure of extravillous trophoblast (EVT) invasion and subsequent malformation of the maternal placental circulation [3, 4]( Fig. 1). Recognizable signs of PE include maternal hypertension accompanied by endothelial and renal dysfunction. Without timely and proper intervention, PE may advance to eclampsia, marked by cerebral edema, epileptic fits, and a heightened risk of mortality for both mother and fetus [5]. Severe early-onset PE (also called placental PE, onset before 34 weeks) poses a significant threat to the health of both the mother and the fetus, placing a considerable burden on families and society as a whole [6]. Notably, placental PE is associated with a greater risk of future health issues, particularly cardiovascular and cerebrovascular diseases, in both mothers and babies [7]. Fetal growth restriction (FGR) is a significant contributor to perinatal mortality and imposes substantial morbidity in neonatal and later life [8]. Unfortunately, effective treatment medications for FGR induced by placenta dysfunction during pregnancy are currently unavailable [9].

Approximately 10% of pregnant women face significant obstetric complications, including preeclampsia and FGR, which can result in maternal and infant morbidity, and in severe cases, mortality [10]. However, the potential off-target effects of many therapeutic interventions given the limited availability of drugs deemed safe during pregnancy. Consequently, the number of drugs licensed for treating pregnancy disorders is extremely limited [11–13]. Numerous studies are underway with the specific aim of targeting the placenta to enhance pregnancy outcomes [14]. A clinical trial using sildenafil citrate for the treatment of severe FGR was halted abruptly due to an elevated neonatal death rate in the treatment group [15]. Therefore, striking a balance between achieving therapeutic efficacy and eliminating the risk of fetal exposure poses a significant challenge in delivering therapeutics to pregnant women.

The objective of this article is to provide a comprehensive review of current targeted treatment strategies and therapeutics delivery methods designed to specifically target the placenta, provide potential solutions for existing problems, and make suggestions for future studies.

## Potential Targeted Treatment Methods for PE and FGR

The current studies on therapeutic interventions that placental-targeted to improve placental function and fetal growth, avoiding any potential adverse off-target effects on the fetus. We summarized the studies about potential placenta-targeted therapeutic strategies in Table 1, and categorized these strategies into cellular and acellular approaches. In Graphical Abstract, Mesenchymal Stem Cells (MSCs) as a cellular approach, acellular approaches including MSCs-derived exosomes and engineering exosomes, nanoparticles-encapsulated therapeutics delivery system, homing peptides-coated therapeutics delivery system and placental-specific gene therapy. These findings establish a foundation for future innovations that have the potential to impact the field of targeted treatments for pregnancy complications significantly.

**Table 1:** The animal model, therapy effects, therapy timeline and frequency, potential mechanism of targeted-placenta treatment

Re.	Therapeutics	Animal model	Potential Mechanism and pathway	Outcomes	The time, method and dose of injection
7	hPMSC	Rat PE; Intravenous injection of L-NAME 50 mg/kg/d from the 10th to 12th GD	Not mentioned	Blood Pressure ↓ Proteinuria ↓ Fetal Weight ↑ placental perfusion ↑ placenta MVD ↑ renal pathology ↑	On GD16; Placenta injection or transplantation; 5 × 10^7^ PMSC in 20 µl 0.9% NaCl
21	MSCs derived from human decidua	Mice PE; Intravenous injection of 10^7 ^Th1 cell on GD10.5 and GD 12.5	Suppression TNF-a in mice.	Blood Pressure ↓ Proteinuria ↓ Fetal Weight ↑ Fetal numbers ↑	On GD11.5 and GD 13.5; Intravenous injection; 10^6^ MSCs suspended in 100 µl PBS
23	HU-MSC	Rat PE; Injection of 2 ml endotoxin solution via jugular vein cannula on GD14; 1µg/kg	Not mentioned	Blood Pressure ↓ Proteinuria ↓ WBC/TNF‑α/ IL-1β ↓ IL-10 ↑	On GD14; Tail vein injection; 2 × 10^6^ cells suspended in 100 µl PBS
24	HU-MSC	Rat PE; Intravenous injection LPS on GD14; 1 µg/kg	Activate PPARγ-related regulatory mechanisms to recover Thl-Th2 balance.	Blood Pressure ↓ 24 h proteinuria ↓ inflammatory cells in the placenta ↓ Fetal Weight ↑ endothelial function ↑	On GD14; Intravenous injection; 2 × 10^6^ cells
47	HU-MSC-Exo	Rat PE; Abdominal injection of L-NAME from GD7 to GD19; 250 mg/kg/d	Not mentioned	Blood Pressure ↓ 24 h proteinuria ↓ Fetal Weight ↑ Fetal numbers ↑	From GD14 to GD19; Abdominal injection; 60-70-80ug/day
59	PLAC1-hIGF-1	Mice IUGR; Induced by uterine artery branch ligation on GD16	Produces measurable IGF-1 RNA expression.	IGF-1↑ Fetal Weight ↑	On GD16; Intra-placental injection; 63ng PLAC1-hIGF-1 DNA
64	T-NP_sisFLT1_	Mice	Silenced sFLT1 in mice.	Silenced sFLT1 in mice; Safe for both the mother and fetus	On GD14 and GD15; Tail vein injection; 2 mg/kg/day
71	ELP-VEGF	Rat PE; Reduced uterine perfusion pressure surgery on GD14	Fused VEGF to elastin-like polypeptide, a biopolymer carrier that does not cross the placental barrier.	Alleviated maternal syndrome; Careful dosing and optimization of the delivery route are necessary.	From GD14 to GD19; Intraperitoneal minipump; 1, 5, and 10 mg/kg/day
72	T-NP_PFKFB3_	Mice	T-NPPFKFB3 upregulated the expression of PFKFB3 without affecting its expression in other important organs.	Fetal Weight ↑ Placenta Weight ↑ Accumulated in mouse placenta; Promoted placental angiogenesis.	On GD12.5, GD14.5, GD16.5; Tail vein injection; 0.025 mg/day
73	SE175	eNOS-/- knockout mice; Pre-pregnancy hypertension and FGR	Targeted SE175 reduced placental expression of 4-hydroxynonenal, cyclooxygenase-1 and cyclooxygenase-2, indicating a reduction in placental oxidative stress.	Fetal Weight ↑	From GD13.5 to GD 15.5; Tail vein injection; 0.44 mg/kg/day
74	siRNA-sFlt1-PAMAM	Rat PE; Intraperitoneal injection TNF-a from GD10 to GD14; 50 ng/day	Not mentioned	sFlt1 secretion↓ Blood Pressure ↓ Proteinuria ↓ Fetal Weight ↑ Placenta Weight ↑	On GD13, GD15, GD17; Tail vein injection; 0.3 mg/kg/day
76	hsiRNAsFLT1-2283/2519	Baboon PE; Induced by ligation of a single uterine artery on GD133	Reduced circulating sFLT1.	Blood Pressure ↓ Proteinuria ↓ Suppressed sFLT1 overexpression; Did not affect pup viability, weight, or ability to thrive.	On GD133; Intravenous injection; 20 mg/kg
77	Ad-hIGF-1	Rabbit IUGR; Induced by mesenteric uterine artery branch ligation on GD21	IGF-1 may play a role in enhancing placental exchange area, function, or metabolism, leading to improved fetal growth.	Fetal Weight ↑ Liver Weight ↑ Musculoskeletal Weight↑	On GD21; Intra-placental injection; 1 × 10^8^ or 1 × 10^9^ plaque forming units (pfu)
78	hIGF-1	Mice IUGR; Induced by uterine artery branch ligation on GD18	Over-expression of IGF-1 impacts placental amino acid transport and contributing to the restoration of fetal weight.	Fetal Weight ↑	On GD18; Intra-placental injection; 1 × 10^8^ plaque forming units (pfu)
80	Ad.VEGF	Sheep FGR; Induced by high caloric intake in early gestation on GD83	Not mentioned	Fetal Weight ↑	On GD83; Intravenous injection; 5 × 10^11^ particles in 10 ml normal saline
81	VEGF-A165	Guinea pig FGR; Induced by Nutrient-restricted from GD0 to GD30	Not mentioned	Fetal Weight ↑ Postnatal weight gain in female pups ↑	On GD34; External surface of the uterine and radial arteries injection; 1 × 10^10^ particles

PE preeclampsia; IUGR intrauterine growth restriction; FGR fetal growth restriction; GD gestational day;↑ increase or ameliorate; ↓ reduce; MVD microvessel density; PBS phosphate buffer solution

### Mesenchymal Stem Cells (MSCs)

Mesenchymal stem cells (MSCs) are multipotent stem cells renowned for their remarkable ability to selectively migrate to injured, ischemic, and hypoxic tissues [16]. Upon transplantation, MSCs exhibit the capacity to home in on damaged tissues, eliciting potent anti-inflammatory, antiviral, anti-apoptotic, and anti-fibrotic effects while concurrently promoting angiogenesis and immune regulation [17]. It is greatly accepted that MSCs can be obtained from many tissues such as the umbilical cord, placenta, bone marrow, and so on, they also can secrete many biomolecules, such as growth factors, cytokines and chemokines, in the manner of autocrine or paracrine and the biomolecules can help their biological activities in consonance to the encompassing microenvironment [18]. The global interest in MSCs has surged owing to their low immunogenicity, robust self-renewal capabilities, and versatile multi-lineage differentiation potential, positioning them as promising candidates for a wide range of clinical applications [19].

Scholars have conducted a series of explorations on the use of MSCs for the treatment of preeclampsia, and they found that MSCs from different sources have distinct advantages [17, 20]. MSCs derived from decidua therapy significantly ameliorated both clinical and histopathological severity in a Th1 cell-induced PE-like mouse model, including a decrease in blood pressure and proteinuria, suppression of glomerulonephritis, and protection of fetoplacental development [21]. The human umbilical cord blood and bone marrow are both excellent sources of MSCs, and the characteristics of MSCs from these two tissues exhibit similarities, however, the quantity of MSCs derived from bone marrow decreases with age, which constraining their clinical application [22, 23]. Human umbilical cord-derived MSCs (HU-MSCs) have a protective effect on endotoxin-induced PE rat models, and this effect is likely elicited through the suppression of inflammatory factors [23]. Transplantation of HU-MSCs dramatically corrected the inflammatory hyperreaction in an LPS-induced rat PE model [24]. MSCs isolated from the placenta also could be exploited for the treatment of hypertension in PE [20], and the clonogenicity and function of human placenta MSCs (hPMSCs) were superior to cord-derived MSCs [25].

It is interesting to note that MSCs can be manipulated in culture to obtain phenotypes that more effectively treat one disease over another [17]. To enhance the function of MSCs, the modified MSCs strive for their effects with their therapeutic paradigm. Wu Di et al. [26] discovered that heme oxygenase-1 (HO-1) gene-modified human placental MSCs (HO-1-PMSCs) enhanced placental vascularization by promoting a balance of pro- and anti-angiogenesis factors. This suggests that HO-1-PMSCs could serve as an alternative treatment for preeclampsia. Liu Yu et al. [7] showed that transplanting HO-1-PMSCs into the placentas of PE rats led to enhanced placental vascular formation, improved placental perfusion, and alleviated symptoms of PE. Considering ethical tissues and potential tumorigenic risks, there are currently no clinical trials registered utilizing MSCs in the treatment of PE and FGR [27]. To clarify the safety of the clinical practice of MSCs, lots of studies have been done and suggested that human-derived MSCs are more resistant than mouse MSCs, there was no genomic instability detected and no tumor-induced after long-term in vivo transfer [28]. Additionally, MSCs are short-lived and do not migrate beyond the lungs, liver, and spleen after intravenous infusion [29]. The placental injection was employed to enhance the targeting and therapeutic efficacy of MSCs, while researchers have found that this approach necessitates invasive procedures, potentially hindering its clinical application. Moreover, partial cells that are transplanted often undergo apoptosis before exerting therapeutic effects, primarily due to poor diffusion of nutrients and oxygen [30, 31], which also brings about barriers to clinical application.

Kabat et al. [32] reviewed many clinical trials about MSCs and they found that the effective dose of MSCs varied widely depending on the disease categories, delivery routes, and the types of MSCs being used. They also found that the relationship between MSCs dosage and efficacy presents an inverted U-shape, once the effect reaches a peak (optimal dosage), increasing the dosage further not only fails to enhance efficacy but may also cause adverse reactions. Although the therapeutic benefits of MSCs have shown promise, the introduction of foreign living cells into the human body is a constant source of concern [33]. Additionally, the varied beneficial effects of MSCs can be attributed to differences in cell characteristics, dosages, and transfusion patterns [25]. Significant variations in the preparation, fitness, and functionality of MSCs can occur depending on the tissue source and culture methods used, these variations should be removed after expansion [28]. Therefore, more researches about MSCs are needed to confirm the molecular mechanisms, optimal timeline, frequency, and dosage to optimize therapeutic outcomes while minimizing potential side effects before its clinical application.

### MSCs-derived Exosomes and Engineering Exosomes

Exosomes are one subtype of EVs with cup-shaped or round and their size ranges from 40 nm to 120 nm. Almost all cells and tissues can secrete exosomes and the secretion is regulated by local environmental factors such as oxygen tension, glucose, and free fatty acid concentration [34, 35]. The presence of maternal EVs in fetal circulation suggests the capability of these EVs to traverse the placental barrier and play a therapeutic role locally within the placenta [36, 37]. Exosomes are known to retain the characteristics of the cells from which they are derived [38]. For example, tumor-derived exosomes may well influence the growth, angiogenesis, invasion, and metastasis of tumors [28]. Comparatively, exosomes derived from MSCs possess properties that make them ideal adjuvants to support and complement other treatment modalities [39, 40]. Here, our discussion is specifically centered on exosomes derived from MSCs.

The research potential of exosomes in treating diseases is significant due to their low immunogenicity, good biocompatibility, and notable homing ability [41, 42]. Particularly, exosomes derived from first-trimester placental mesenchymal stem cells (PMSCs) exhibit a novel homologous homing function, enhancing endothelial cell migration and vascular tube formation [43]. Exosomes are also capable of transferring their contents, such as RNAs and proteins, to other cells, thereby regulating the biological function of the target cell [44]. Numerous studies have affirmed the crucial role of exosomes in spiral artery remodeling and angiogenesis, processes highly associated with PE [35, 45, 46]. Interestingly, the quantity of exosomes throughout gestation is higher in PE, compared to normal pregnancies, and some specific contents in exosomes are markedly changed [35]. These exosomes from PE may contain biomarkers and molecules that reflect the stressed state of the placenta and other maternal tissues, such as inflammatory cytokines, angiogenic factors, and stress-related miRNAs [35, 46]. Whereas, the exosomes used to treat disease are normal cell-derived. For example, exosomes derived from human umbilical cord MSCs contain angiogenic factors that promote placenta angiogenesis and alleviate symptoms of PE, and this effect has a dose-dependent manner [47]. Exosomes can be modified to carry specific therapeutic payloads, such as siRNA, miRNA, or specific proteins that can directly interact with and modulate the molecular pathways implicated in PE [41].

With the emergence and advancement of precision medicine, scholars have investigated engineering exosomes to enhance their therapeutic efficacy and targeting precision, including genetic manipulation of producer cells and surface modification of exosomes. However, challenges exist, as genetic engineering is dependent on transfection efficiency and cell types, modifying native exosomes chemically can substantially alter their surface structure which may affect their stability [48]. Multiple engineering approaches, including physical and chemical modifications, as well as the direct encapsulation of macromolecules into exosomes, have been employed to enhance exosomal targeting, nonetheless, these techniques encounter challenges associated with low efficiencies [49–51]. To address these limitations, researchers have utilized phage display technology to identify peptides that are capable of anchoring cargo and targeting moieties to exosomes. These peptides simplify the process of loading and functionalizing exosomes, making exosomes a targeted treatment option. For instance, employing targeted peptides, like Myd88, to load synthetic eukaryotic vesicles (SyEV) has enhanced their therapeutic potential in outer membrane vesicle-induced macrophages without causing severe side effects [52]. Additionally, the modification of human umbilical cord-derived exosomes with the targeting peptide HSTP1 enables precise treatment for activated hepatic stellate cells (aHSCs) within complex liver tissue [40]. Studies have also confirmed that exosomes derived from cells subjected to hypoxia pretreatment can enhance angiogenic function and therapeutic effects [45, 53]. However, the limitation that MSCs cannot proliferate indefinitely poses a challenge to the mass production of MSC-derived exosomes. In addressing this, researchers transfected the MYC gene into MSCs, revealing that MYC-transfected MSCs exhibited faster growth, reduced adhesion, decreased aging, and the exosomes they produced with similar therapeutic efficacy as those produced by untransformed MSCs [54]. While MYC-transfected cells may induce alterations in MSC phenotype, resulting in the loss of characteristic MSC properties or impacting cellular genomic stability [55]. MYC as an oncogene may trigger mutations or chromosomal instabilities, potentially jeopardizing the safety of the therapy [56]. The enduring consequences of MYC-transfected MSCs remain uncertain, more research should be done to clarify its long-term safety and to explore scalable methods for the mass production of stem cell exosomes in the future.

Numerous studies have explored the promising treatment of MSC-derived exosomes in the nervous system, yielding encouraging results [33]. While there is no doubt about the potential of exosomes in treating PE, their clinical application requires further investigation and confirmation. This includes an in-depth exploration of mechanisms, safety considerations, therapeutic effects, delivery methods, and optimal dosage for exosomes in the treatment of preeclampsia.

### Nanoparticle-encapsulated Therapeutics Delivery System

The field of engineered nanoparticles (NPs) is rapidly expanding in industrial activity, with applications developed for various purposes. In the realm of medicine, NPs have been explored for drug delivery to specific tissues, especially in the treatment of various cancers [57]. Zhang et al. [10] discovered that chondroitin sulfate A-binding peptide (CSA-BP)-conjugated lipid-polymer nanoparticles exhibited high drug-carrying capacity, stability, and efficient delivery to the placenta. This breakthrough finding opens up significant opportunities for targeted therapeutic strategies in managing pregnancy complications. However, it is crucial to note that evidence, particularly from in vivo rat studies, suggests that certain nanoparticles, such as gold nanoparticles (NPs), can cross the placenta barrier and accumulate in the fetus [57]. Yang et al. [58] used gold NPs to study placental development and observed that intravenous injection of gold NPs resulted in higher fetal accumulation when administered early in mouse gestation (before E11.5) compared to later in gestation.

Ellah et al. [59] developed a nanostructure delivery system, which complexed with IGF-1 and trophoblast-specific promoter PLAC1, as targeted therapy for FGR, and they found that the birth weight in mice could be restored to normal after intra-placental injection of PLAC1-IGF-1 nanoparticles, and the therapy is not detrimental to normal placental morphological development. A novel approach involved engineered NPs covered with a synthetic placental chondroitin sulfate-A binding peptide (plCSA) or single-chain antibody fragments against the EGF receptor, targeting specifically trophoblast cells in the human and mouse placenta in vitro, importantly, this targeting did not extend to the decidua, fetus, or any maternal tissues after intravenous injection [60, 61]. The following study confirmed increased placental function in human placental trophoblast cells and placental explants after transfection with PLAC1-IGF-1 nanoparticles [62]. However, it remains uncertain whether placental targeting of IGF-1 can be achieved through peripheral administration of NPs coated with tumor-homing peptides in a diblock configuration.

An important consideration for placenta-targeted NPs is the gestational age, as placenta physiology and transport changes throughout gestation. Ho et al. [63] demonstrated that modifying NPs with polyethyleneimine (PEI) may be a viable platform for placenta-specific delivery depending on the gestational age. To confirm the fetus safety forward, the following research designed trophoblast-targeted NPs to treat PE mice which showed no toxic effects to injected mice or fetuses, and pups’ birth weights were comparable to those treated with untargeted NPs [64]. Additionally, recent achievements in placenta trophoblast-targeted NP drug delivery have been made through PLGA microparticles controlling heterogeneous human placental matrix release to modulate angiogenesis [65]. For a more active and intriguing approach to targeting the placenta, NPs can be surface-modified with ligands such as peptides, antibodies, or aptamers that specifically bind to cell surface receptors on the placenta [66]. Hence, further research is needed to better understand diseases of pregnancy and develop targeted approaches to treat placental dysfunction diseases.

In general, the use of nanoparticles for the treatment of placental diseases raises important questions and concerns due to their potential to cross the placental barrier. It is essential to carefully weigh the therapeutic advantages against potential adverse effects. Further research is also needed to thoroughly investigate the dose, treatment timeline frequency, and efficacy when nanoparticles are applied to treat placental diseases.

### Homing Peptides-coated Therapeutics Delivery System

Targeted therapy is at the forefront of contemporary medical research, with growing interest among researchers. Studies have revealed that the placenta shares many features with solid tumors, such as rapid cell proliferation, the production of growth-associated cytokines, and the ability to evade immune surveillance [67]. Homing peptides, which are polypeptides developed using phage display technology, can specifically recognize and bind to certain receptors or markers. These peptides can accumulate drugs at the sites of symptoms, minimizing adverse effects on normal cells and tissues [67, 68]. Moreover, research suggested that peptides with the ability targeting to tumors could also be applied to treat obstetrical diseases originating from the placenta [69].

Elastin-like Polypeptides (ELPs) are specifically designed to avoid active transport mechanisms and can be tailored to incorporate therapeutics, acting as a non-immunogenic drug delivery platform. These properties make ELPs suitable for treating maternal diseases during pregnancy. George et al. [70] conducted intravenous injection of ELPs into rats on gestational day 14 to evaluate their potential for maternal drug delivery. They observed that ELPs predominantly accumulated in maternal tissues and the placenta, with minimal presence in the pups. Additionally, high levels of soluble fms-like tyrosine kinase-1 (sFlt-1), a known precursor to preeclampsia, sequesters VEGF, leads to endothelial dysfunction and severe hypertension. A study suggested that ELP-VEGF has the potential to treat PE with minimal fetal exposure, however, it did not study the dose and the appropriate delivery route, and further research should be done to evaluate those [71]. King et al. [67] intravenous injected tumor-homing peptides (CGKRK and iRGD), labeled with 5(6)-carboxyfluorescein (FAM) into pregnancy mice, found a significant enrichment in placental tissue compared to other organs, immunochemistry confirmed that the honing peptides attached to the endothelium of non-remodeled spiral arteries and the endovascular trophoblast lining remodeled arteries. The study also suggested that intravenous injection liposomes containing IGF-2 attached homing peptides enhanced placental but not fetal weight, this effect was more pronounced than those elicited by IGF-2 alone or IGF-2 in liposomes without homing peptides. Similarly, a study found that injecting liposomes intravenously, which contained PFKFB3 overexpression plasmids modified with the placental homing peptide CGKRK, promoted placental angiogenesis and increased both fetal and placenta weights of the mice without adversely affecting other vital organs [72]. Furthermore, Cureton et al. [73] discovered that the placental-specific peptide NKGLRNK could deliver vasodilator directly to the uteroplacental vasculature in both mice and human placenta in vivo and in vitro. Notably, these peptides did not accumulate in any other maternal or fetal tissues.

Peptide-directed targeting provides a new platform for placenta-specific treatment in PE and FGR. However, the dose, treatment timeline, and frequency are still uncertain, more researches are required before these homing peptide-modified therapeutics can be applied clinically. The prospect of using such targeted treatments for placental dysfunction diseases holds significant potential.

### Targeted-Placental Gene Therapy

Gene therapy is a crucial method for treating diseases using DNA or RNA, either directly introduced or encapsulated with plasmids, viruses, bacteria, etc. Transient gene regulation using siRNA or mRNA also holds ample opportunities for treating placental disorders during pregnancy. Recent studies have demonstrated the use of siRNA to treat PE by inhibiting the secretion of sFlt-1 from trophoblasts, showcasing the potential of siRNA-mediated knockdown of sFlt-1 in PE treatment [59]. In a similar study, sFlt-1 siRNA encapsulated within PAMAM dendrimers showed efficacy in inhibiting sFlt-1 secretion, lowering maternal hypertension, and prolonging pregnancy in PE rats [74]. Associations between higher sFLT-1 levels and increased expression of miR-195-5p in PE women have been identified [75]. Using PEG-PLA nanoparticles conjugated with placental CSA binding peptide (P-CSA-BP), a novel placenta-specific sFLT1 siRNA delivery system was developed, effectively silencing sFLT1 in treated mice and ensuring safety for both the mother and fetus [64]. In a baboon PE model induced by uterine artery ligation, a single dose of siRNAs suppressed the expression of sFLT1, and improved clinical signs of PE, such as maternal hypertension and proteinuria [76]. Moreover, intra-placental injection of the human IGF-1 gene, driven by a CMV promoter, into rabbit/mouse Intrauterine Growth Restriction (IUGR) models showed minimal gene transfer to fetuses or maternal organs and prevented cardiac dysfunction caused by excessive FGR in offspring while restoring weight [77–79]. . Carr et al. [80] established a sheep model of FGR induced by high caloric intake in early gestation, where the injection of adenovirus containing the VEGF-A165 gene (Ad. VEGF) improved fetal growth without affecting normal fetuses significantly. Additionally, the VEGF-A165 gene applied to the surface of the uterine artery in a guinea pig FGR model improved birth weight, with a more pronounced effect in female pups [81]. Another study selectively delivered VEGF DNA to the placental basal plate using microbubble carriers after intravenous injection in early pregnancy, correcting impaired uterine artery remodeling [82].

A substantial amount of noncoding RNAs, particularly microRNAs (miRNAs) and long noncoding RNAs (lncRNAs) has gained significant attention due to their biological impact on PE [83]. MiRNAs regulate gene expression post-transcriptionally, and their altered expression has been observed in PE [4, 84]. Placental miRNAs have been detected in the blood of pregnant women, showcasing their potential as biomarkers [85]. For instance, the concentration of placental miRNA-141 in maternal plasma increases with gestational age [86], and placenta-specific miRNA-517 A, released from chorionic villous trophoblasts, circulates in maternal blood and may influence maternal tissues during pregnancy [87]. MiR-26a-5P has been identified in the urine of preeclamptic patients with proteinuria [88], while elevated levels of miR-195-5P in preeclamptic pregnant women correlate with sFlt-1 [75]. The regulatory role of miRNAs extends to trophoblastic cells, where miR-144 influences the proliferation and apoptosis of these cells by targeting the PTEN signaling pathway [89]. Furthermore, a set of placenta-specific miRNAs (miR-126-3P, miR-410-5P miR-515-5P, -516b, -516a-5P, -518b, -519d, -520 h, -520a-5P, -525-5P, -526b, and − 1323) is significantly elevated in the plasma of PE patients compared to normal pregnant women [90–92]. Long non-coding RNAs (lncRNAs), which act as coregulators or complementary binding molecules to regulate gene expression, may also play a role in PE. The upregulation of lncRNA PSG10P, a noncoding pseudogene, has been observed in PE placentas [93]. Above all, these noncoding RNAs may offer potential therapeutic targets for the treatment of PE and FGR.

However, translating maternal gene therapy to clinical applications is indeed a complex process. The EVERREST Project, funded by the European Commission, has set out to conduct a clinical trial aimed at evaluating the safety and efficacy of maternal uterine artery Ad.VEGF gene therapy for severe early-onset FGR [94]. While intraplacental gene therapy is more invasive compared to administering oral medication, it holds the potential advantage of precisely targeting vasoactive changes to the maternal uteroplacental circulation, thereby minimizing systemic effects. Appropriate gene therapy also depends on the development of safe and efficient gene delivery systems to target cells in the body, this could include viral vectors, lipid nanoparticles, and other methods.

Before gene therapies for placental disorders, such as FGR and PE, can be considered for clinical use, more comprehensive researches are needed. These include conducting additional preclinical studies, assessing long-term outcomes, understanding potential side effects, and ensuring the safety and efficacy of the gene therapy approaches. Well-designed clinical trials are essential to validate the findings and determine the feasibility of translating these therapeutic strategies into practical and effective treatments for pregnant individuals experiencing placental dysfunction.

## Conclusions

The ethical dilemma inherent in treating diseases during pregnancy underscores the need to carefully balance the therapeutic benefits for the mother and the potential risks for the fetus. Recent research explored placenta-specific targeting as a therapy option that holds promise for novel treatments of placental disorders, particularly PE and FGR in obstetrics. The timeline of placenta-targeted therapy all after gestational day 11.5 in mouse and/or rat models, equivalent to mid to late pregnancy, the frequency and the dose of treatment still uncertain. The research about placenta-targeted treatments for PE and FGR is still in the experimental, besides the distinct differences in placentation and maternal-fetal interaction between rodents/primates and humans/nonhuman primates, advanced research could utilize primate models to investigate various aspects of placenta-targeted therapy. This includes investigating the appropriate dose, timeline, therapeutic frequency (single time or multiple), and the effects of such strategies in preventing placental dysfunction diseases. Furthermore, it is crucial to thoroughly study the underlying mechanisms and safety of these therapies before they can be implemented in clinical practice.

## Data Availability

Not applicable.
